# Optimization of Bonding Behavior of Crumb Rubber-Modified (CRM) Asphalt for Sustainable High-Friction Surface Treatment (HFST) Applications

**DOI:** 10.3390/ma19173619

**Published:** 2026-08-26

**Authors:** Abdallah Aboelela, Magdy Abdelrahman

**Affiliations:** Department of Civil, Architectural, and Environmental Engineering, Missouri University of Science and Technology, Rolla, MO 65409, USA; a.aboelela@mst.edu

**Keywords:** binder bonding strength (BBS), high-friction surface treatment (HFST), crumb rubber-modified (CRM) asphalt, moisture resistance, friction retention, rhyolite aggregate

## Abstract

Binder bonding strength (BBS) is critical to aggregate retention and friction performance in high-friction surface treatment (HFST). Although epoxy resin is traditionally used, asphalt-based binders offer a sustainable alternative; however, their bonding behavior and influence on polishing performance remain insufficiently understood. This study investigated the evolution and optimization of BBS in crumb rubber-modified (CRM) asphalt for rhyolite-based HFSTs. BBS was measured under dry and wet-conditioned states for two CR contents (10% and 15%), two CR types (cryogenic and ambient), two interaction temperatures (170 and 200 °C), and interaction times from 10 to 240 min. HFST performance was assessed using the British pendulum tester (BPT), dynamic friction tester (DFT), and circular track meter (CTM) under accelerated polishing. CR modification reduced dry BBS relative to the base binder but substantially improved moisture resistance: wet conditioning reduced base-binder BBS by 23.8%, versus 5.8–9.8% for CRM binders. BBS evolution was temperature-dependent. At 170 °C, BBS decreased and gradually recovered through 240 min, while binders prepared at 200 °C peaked at 120 min before declining. Type III factorial ANOVA identified CR content as the dominant factor affecting BBS, with interaction temperature, interaction time, and their combined effects also being significant. Despite lower BBS, 15% CRM binders generally retained higher HFST friction performance than 10% binders after accelerated polishing. The moderate relationship between dry BBS and coefficient of friction (COF) loss (R^2^ = 0.68) confirmed that BBS alone could not predict HFST durability, while a stronger BPN-COF loss correlation (R^2^ = 0.79) confirmed consistent rankings across friction test methods. Cryogenic CRM prepared at 200 °C for 120 min provided the best balance among bonding development, rheology, and friction retention. These findings demonstrate that while bonding strength alone does not govern HFST durability across interactions, its evolution with interaction time exerts a significant effect on friction retention within a given interaction condition, highlighting interaction-time optimization as a critical parameter for developing sustainable CRM-based HFSTs.

## 1. Background

Maintaining adequate pavement friction is essential for reducing crashes at high-risk locations [1,2]. High-friction surface treatment (HFST) improves skid resistance by embedding polish-resistant aggregate in a high-strength binder [3,4]. Calcined bauxite and epoxy are traditionally used in HFST systems; however, the high cost and limited availability of calcined bauxite have encouraged the use of alternatives such as rhyolite [5,6,7,8,9]. Similarly, the need for a more flexible and pavement-compatible binder, particularly for aged or distressed asphalt surfaces, has promoted asphalt as an alternative to epoxy [9,10,11].

HFST performance depends on binder bonding strength (BBS), stiffness, and elasticity, which govern aggregate retention and resistance to traffic-induced stresses. Aggregate–binder bonding is particularly critical because inadequate interfacial capacity can lead to premature chip loss, especially on aged or distressed pavements [9,12,13,14,15,16,17]. Accordingly, Li et al. (2024) and Shen et al. (2025) improved epoxy performance through modified formulations that enhanced bonding and aggregate retention [17,18]. For asphalt-based systems, Roshan et al. (2025) reported that modified mastics and emulsions improved bonding, aggregate retention, and friction durability relative to conventional binders [19].

Among the available approaches for enhancing asphalt binders, crumb rubber (CR) modification has drawn particular attention [20,21,22]. CR modification enhances the viscoelastic response of asphalt by increasing binder stiffness and elasticity, while simultaneously providing a sustainable outlet for waste tires and reducing reliance on virgin materials [20,21]. Building on these benefits, Aboelela et al. (2025) evaluated CRM asphalt as a sustainable HFST binder under different CR contents and interaction conditions [11]. Their results showed that CRM rheology strongly influenced polishing performance: 15% CR binders provided greater stiffness and elasticity, improving friction retention and limiting aggregate movement, whereas 10% CR binders better preserved macrotexture [11].

Several studies have optimized CRM asphalt for mixture performance by controlling CR–binder interaction conditions [22,23,24]. Abdelrahman and Carpenter (1999) identified rubber-particle swelling and degradation through devulcanization and depolymerization as competing mechanisms governed by temperature, time, particle size, and material source [22]. Ragab et al. (2013) found that interaction at 190 °C and 50 Hz promoted a three-dimensional internal network that improved stiffness and elasticity, whereas interaction at 220 °C under high shear caused property deterioration [23].

Competing swelling and degradation mechanisms may explain the inconsistent bonding results reported for CRM binders [25,26,27,28,29]. Lv et al. (2022) found that CR reduced BBS by decreasing the effective binder–aggregate contact area, although the corresponding mixtures showed improved moisture resistance, indicating that lower BBS does not necessarily imply poorer durability [25]. Conversely, Mohamed et al. (2022) reported that in reclaimed asphalt rejuvenated with CRM binder, CR-asphalt interaction enhanced cohesion and bonding strength through physicochemical and rheological changes [27].

Although CRM asphalt bonding has been investigated for asphalt-mixture applications, limited attention has been given to how bonding behavior evolves with CR type, CR content, interaction temperature, and interaction time. Previous studies commonly evaluated BBS under fixed conditions, leaving the effects of the interaction process on bonding development insufficiently understood. In HFST applications, Aboelela et al. (2025) primarily examined the influence of CRM rheological properties on polishing performance without directly characterizing bonding evolution or its relationship with aggregate retention and friction loss [11]. Therefore, a critical gap remains in linking CRM interaction conditions, bonding behavior, and HFST performance.

Therefore, the objective of this study is to optimize the bonding behavior of CRM asphalt binders based on their interaction parameters and to evaluate the influence of this optimization on the performance of HFST systems. These objectives were achieved as follows:

- The bonding behavior of CRM asphalt binders was optimized by evaluating BBS under dry and moisture-conditioned states as a function of CR type, CR content, interaction temperature, and interaction time, supported by concurrent rheological characterization to interpret the observed bonding trends.
- The influence of this optimized bonding behavior on HFST performance was evaluated through aggregate retention and polishing performance testing of rhyolite-based systems.

**Figure 1:**
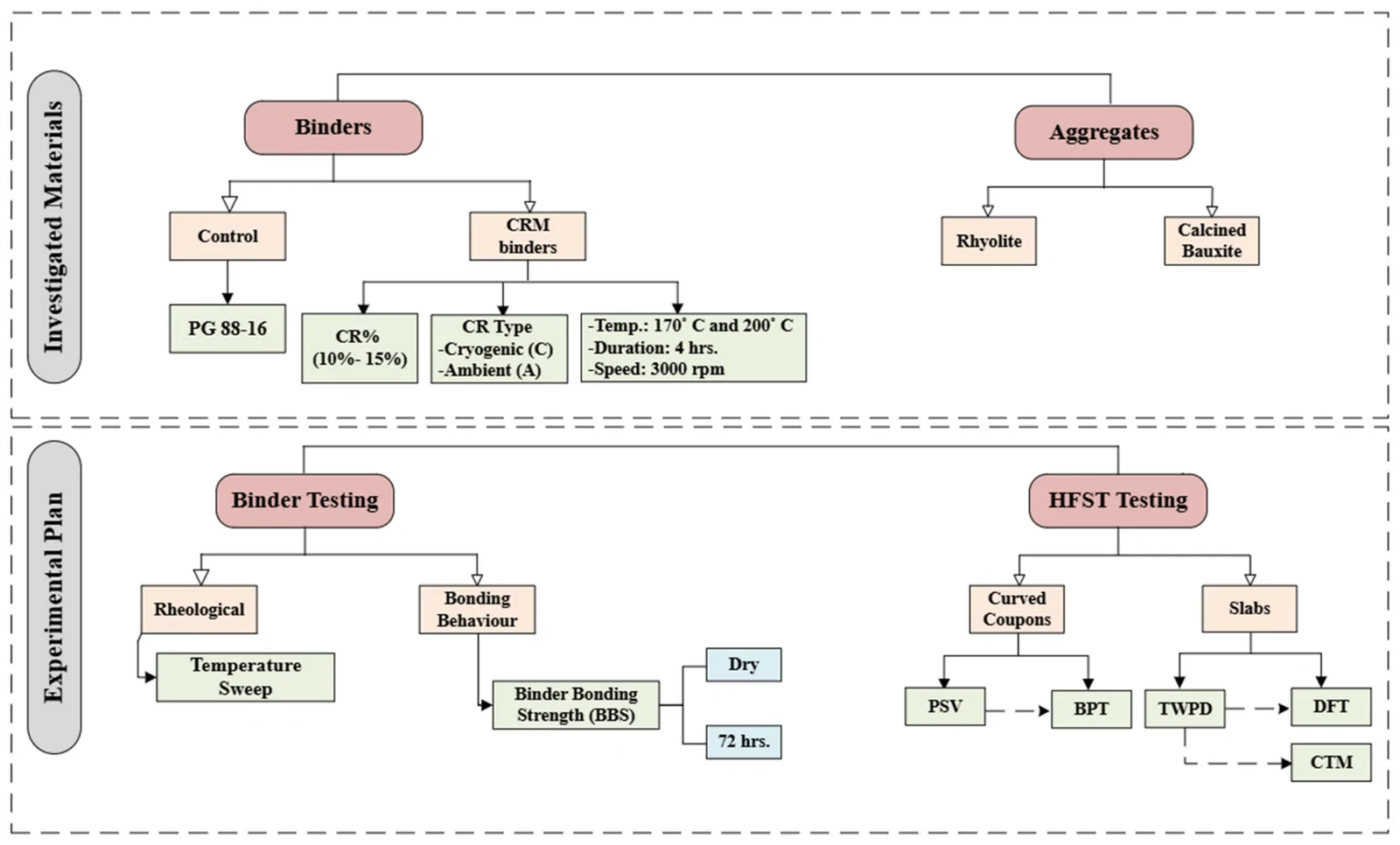
Methodological Framework Illustrating the Investigated Materials, Characterization Tests, and HFST Performance Evaluation Program.

Figure 1 presents the methodological framework adopted in this study, including the raw materials, CR–bitumen interaction conditions, and the characterization and performance testing programs.

## 2. Materials and Methods

### 2.1. Materials

#### 2.1.1. Raw Materials

One hard asphalt binder provided by New Frontier Materials Inc. (Maryland Heights, MO, USA) was used throughout this investigation. As shown in Table 1, the binder exhibited a penetration value of 1.94 mm at 25 °C and performance grade PG 88-16, indicating a highly stiff material. Such a binder was selected to enhance aggregate retention by providing stiffness characteristics comparable to those of epoxy resin, consistent with recommendations reported in the HFST literature [10,11].

Two aggregate types, calcined bauxite and rhyolite, were evaluated in this study. Calcined bauxite is characterized by a high alumina content exceeding 85%, which contributes to its exceptional hardness, polishing resistance, and long-term skid resistance. In contrast, rhyolite is a silica-rich aggregate with higher SiO_2_ content. While rhyolite has demonstrated promising friction performance in previous HFST studies, its high silica content may reduce its affinity with asphalt-based binders and potentially weaken aggregate–binder adhesion. Table 2 summarizes the physical properties of the investigated aggregates. Both aggregates exhibited excellent abrasion resistance, as indicated by their low Los Angeles Abrasion (LAA) values of 16% and 17% for calcined bauxite and rhyolite, respectively. In addition, calcined bauxite exhibited higher specific gravity than rhyolite. A single aggregate gradation was used for both materials, satisfying the Missouri Department of Transportation (MoDOT) requirements for HFST applications.

#### 2.1.2. Preparation of CRM Asphalt

**Figure 2:**
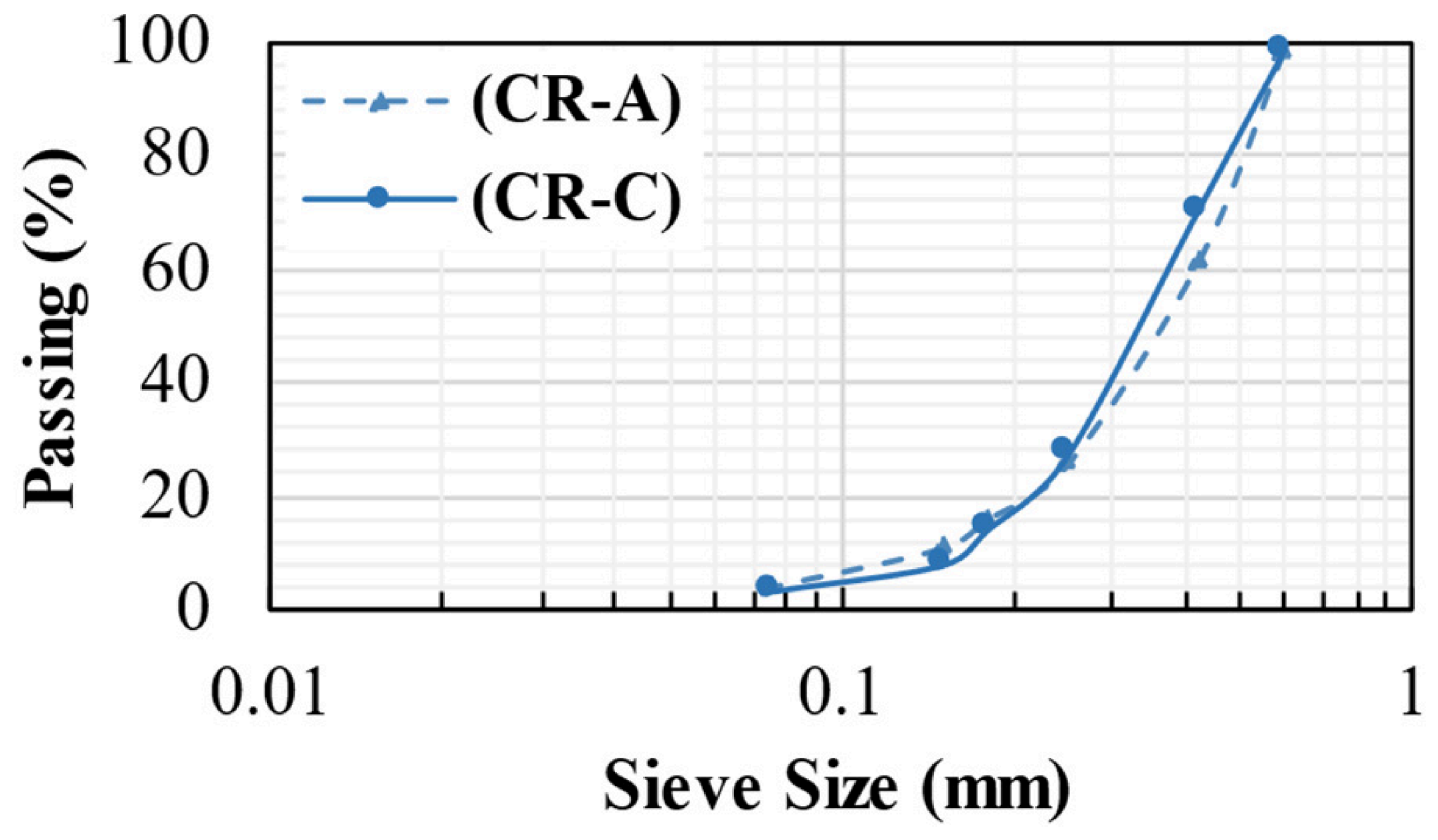
Particle-Size Distributions of the Investigated Cryogenic and Ambient Crumb Rubber.

Two CR types supplied by Lehigh Technologies Inc. (Tucker, GA, USA) were used in this study. Both materials consisted of micronized rubber powder produced from recycled truck tires but differed in their manufacturing processes. One was produced through cryogenic grinding and was designated CR-C, whereas the other was produced through ambient grinding and was designated CR-A. These production methods result in differences in particle morphology and surface characteristics, which can influence CR dispersion and interaction with the asphalt binder [38,39]. Figure 2 presents the particle-size distributions of the investigated CR materials.

CRM binders were prepared using a Ross high-shear mixer. Approximately 1500 g of the base asphalt binder was weighed and conditioned in a forced-draft oven at the target interaction temperature for 3 h to achieve thermal equilibrium. A preheated gallon-size heating mantle was used to maintain a constant temperature throughout the interaction process. The conditioned binder was initially mixed at 750 rpm for 15 min until the target temperature was re-established. The mixing speed was then gradually increased to 3000 rpm, and the CR was slowly added to promote uniform dispersion and minimize particle agglomeration.

The interaction process was conducted at two temperatures, 170 and 200 °C, using CR contents of 10% and 15% for both CR-C and CR-A. To evaluate the evolution of the CR–binder interaction, samples were collected after 10, 30, 60, 120, and 240 min. Accordingly, the experimental program enabled the effects of CR type, CR content, interaction temperature, and interaction time on the bonding and rheological properties of the CRM binders to be evaluated.

### 2.2. Methods

#### 2.2.1. Temperature Sweep Test

A temperature sweep test was conducted on the base asphalt and CRM asphalt binders to evaluate their rheological properties using a Dynamic Shear Rheometer (DSR) (Anton Paar MCR 302e, Anton Paar GmbH, Graz, Austria). All binders were tested in the unaged condition using a 25-mm parallel plate geometry. A sample thickness of 2 mm was adopted for all specimens to ensure consistency across the tested binders and to accommodate the presence of CR particles within the CRM asphalt binders. A constant strain amplitude of 12% and a loading frequency of 10 rad/s were applied throughout the test. The temperature sweep was conducted over a temperature range of 64 °C to 88 °C at 6 °C intervals.

#### 2.2.2. Pull-Off Test

**Figure 3:**
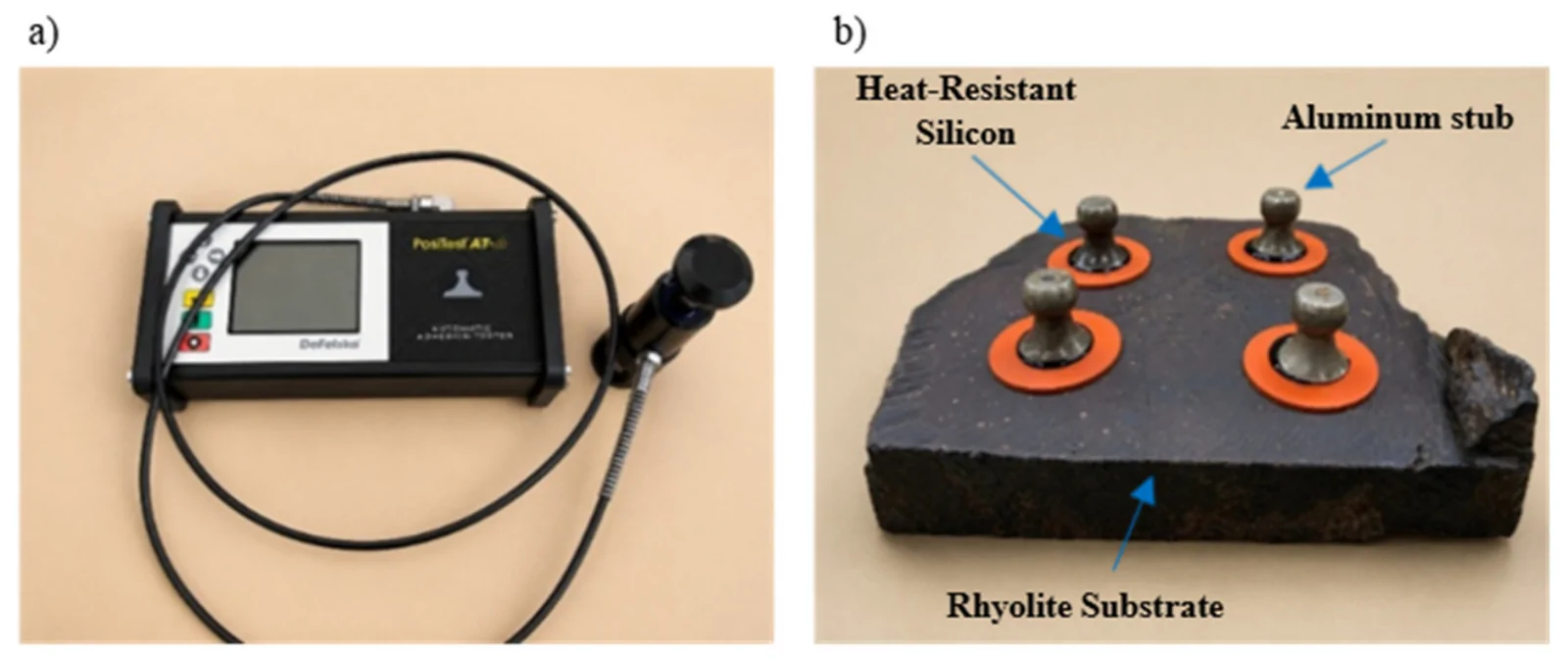
Components of the BBS Testing System: (**a**) PosiTest AT-A Adhesion Tester and (**b**) Prepared Rhyolite Substrate with aluminum stubs.

Binder bond strength (BBS) was evaluated in accordance with AASHTO T 361 using a PosiTest AT-A adhesion tester (DeFelsko Corporation, Ogdensburg, NY, USA) (Figure 3a) [40]. Rhyolite aggregate slabs were used as the substrate (Figure 3b), and 20-mm-diameter aluminum stubs were employed to apply the tensile load. A heat-resistant silicone ring was used to prevent binder flow during specimen preparation.

The substrates were ultrasonically cleaned at 60 °C for 60 min, and both substrates and stubs were oven-conditioned at 150 °C for at least 30 min. Binders and substrates were then heated to the required application temperature of 150–180 °C. The heated binder was placed within the silicone mold, contacted with the aluminum stub for approximately 10 s, and pressed onto the substrate. Pull-off testing was conducted at 0.7 MPa/s. Dry specimens were conditioned at room temperature for 24 h, whereas moisture-conditioned specimens were conditioned for 1 h, immersed in water at 40 °C for 72 h, and equilibrated at laboratory temperature for 1 h before testing.

#### 2.2.3. Preparation of HFST Specimens

**Figure 4:**
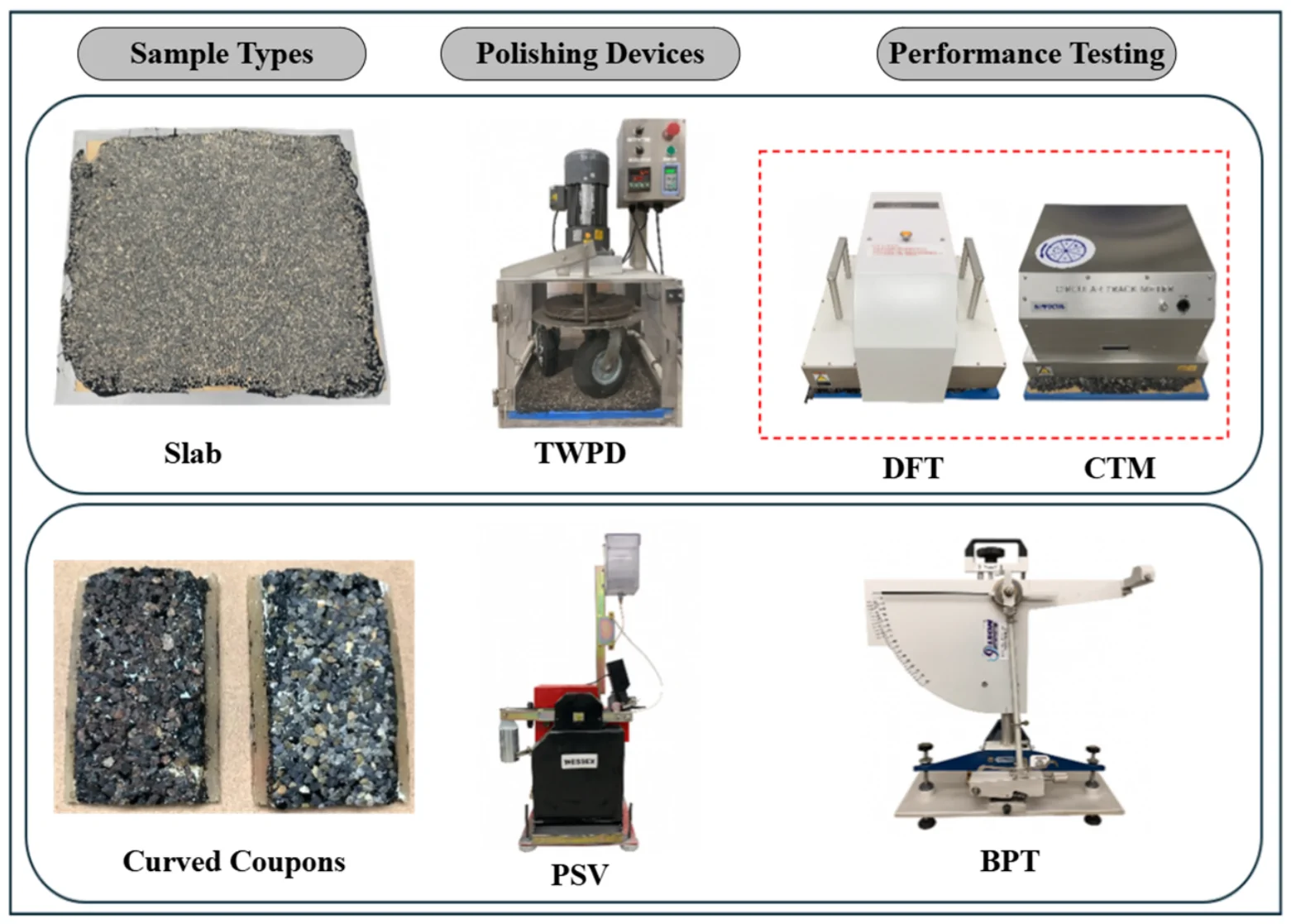
Experimental Procedure for Accelerated Polishing and Friction Evaluation [11].

HFST specimens were prepared in the form of curved coupons and slab samples as shown in Figure 4. Two binder application rates, 0.38 and 0.44 gal/yd^2^, were used for all investigated binder–aggregate combinations. Prior to specimen preparation, the binders and aggregates were heated to their respective application temperatures to ensure proper coating and workability. For the slab specimens, a wooden panel measuring 20 in. × 20 in. was used as the substrate. The binder was uniformly applied to the substrate at the designated application rate, followed by the distribution of aggregate particles to ensure complete surface coverage and consistent aggregate embedment. Three replicate coupon specimens and two replicate slab specimens were prepared for each test condition to ensure the repeatability of the experimental results.

#### 2.2.4. British Pendulum Tester (BPT)

The BPT was used to assess HFST microtexture and skid resistance in accordance with AASHTO T 278 [41]. Testing was conducted on curved coupons using a 1.25-in.-wide rubber slider, and the average of at least five measurements was reported as the British Pendulum Number (BPN). The specimens were then polished using the Polished Stone Value (PSV) apparatus in accordance with AASHTO T 279 [42], with a steel wheel rotating at 320 rpm under a load of 391.44 ± 4.45 N. This procedure enabled evaluation of friction retention and aggregate durability under accelerated polishing. Figure 4 presents the curved coupon specimen, the PSV apparatus used for accelerated polishing, and the BPT used for friction measurements.

#### 2.2.5. Three-Wheel Polishing Device (TWPD)

To evaluate the long-term skid resistance and simulate the effects of field traffic wear on the HFST slab specimens, an accelerated laboratory conditioning process was performed using a three-wheel polishing device (TWPD) in accordance with AASHTO PP 104 [43]. The TWPD induces accelerated surface polishing via a rotating carriage equipped with three pneumatic rubber tires. During operation, a constant combined vertical load of 105 lb was exerted by the wheel assembly onto the test slabs, while the inflation pressure of each tire was maintained at 35 ± 5 psi. The specimens were subjected to a total of 140k polishing cycles.

#### 2.2.6. Dynamic Friction Tester (DFT)

The DFT was used to evaluate the friction performance of the HFST slab specimens under wet conditions in accordance with ASTM E1911 [44]. The test measures the coefficient of friction (COF) by recording the frictional resistance generated as a rotating disc equipped with rubber sliders decelerates upon contact with the specimen surface. Friction measurements were obtained before polishing and after 12k, 30k, 70k, and 140k polishing cycles using the TWPD. The COF values were determined at speed of 20 km/h.

#### 2.2.7. Circular Track Meter (CTM)

The CTM was used to evaluate the surface macrotexture of the HFST slab specimens in accordance with ASTM E2157 [45]. The test determines the Mean Profile Depth (MPD) by measuring the surface profile along a circular path using a laser displacement sensor. Macrotexture measurements were conducted before polishing and at the same polishing intervals used in DFT measurements. Figure 4 presents the prepared HFST slab specimens, the TWPD, the DFT, and CTM used for polishing and performance evaluation.

## 3. Results and Discussion

### 3.1. Analysis of Rheological Properties

**Figure 5:**
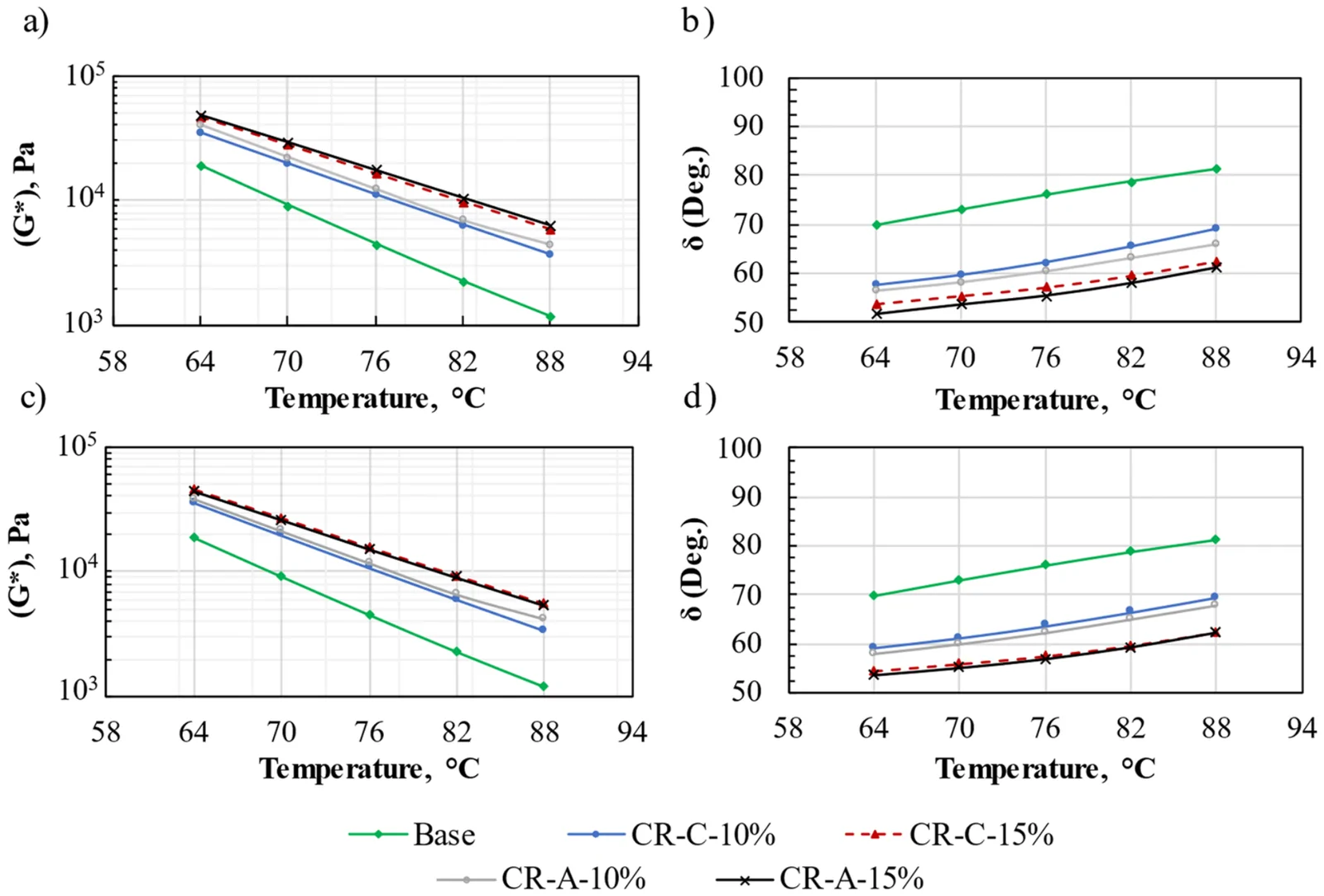
Temperature-Dependent Rheological Properties of Investigated CRM Binders: (**a**) G* for 170 °C-Based CRM, (**b**) δ for 170 °C-Based CRM, (**c**) G* for 200 °C-Based CRM, (**d**) δ for 200 °C-Based CRM.

Figure 5 presents the temperature-sweep results after 240 min of interaction. The base binder exhibited the lowest G* and highest δ, confirming a softer and more viscous response than the CRM binders. CR incorporation increased stiffness and elasticity across the investigated temperature range, and increasing CR content from 10% to 15% consistently increased G* and reduced δ. At 88 °C, for example, G* increased from 3.71 to 5.85 kPa for the cryogenic binders prepared at 170 °C and from 3.40 to 5.56 kPa for those prepared at 200 °C. Thus, CR content produced the strongest rheological modification.

Interaction temperature also influenced the long-term rheological response. After 240 min, binders prepared at 170 °C generally retained higher G* and lower δ than their corresponding 200 °C binders, particularly for ambient CR. For example, CR-A-170-15% exhibited a G* of 6.35 kPa at 88 °C compared with 5.45 kPa for CR-A-200-15%. However, neither CR type consistently produced superior rheological properties under all conditions, indicating that the effect of CR type depended on CR content and interaction temperature.

**Figure 6:**
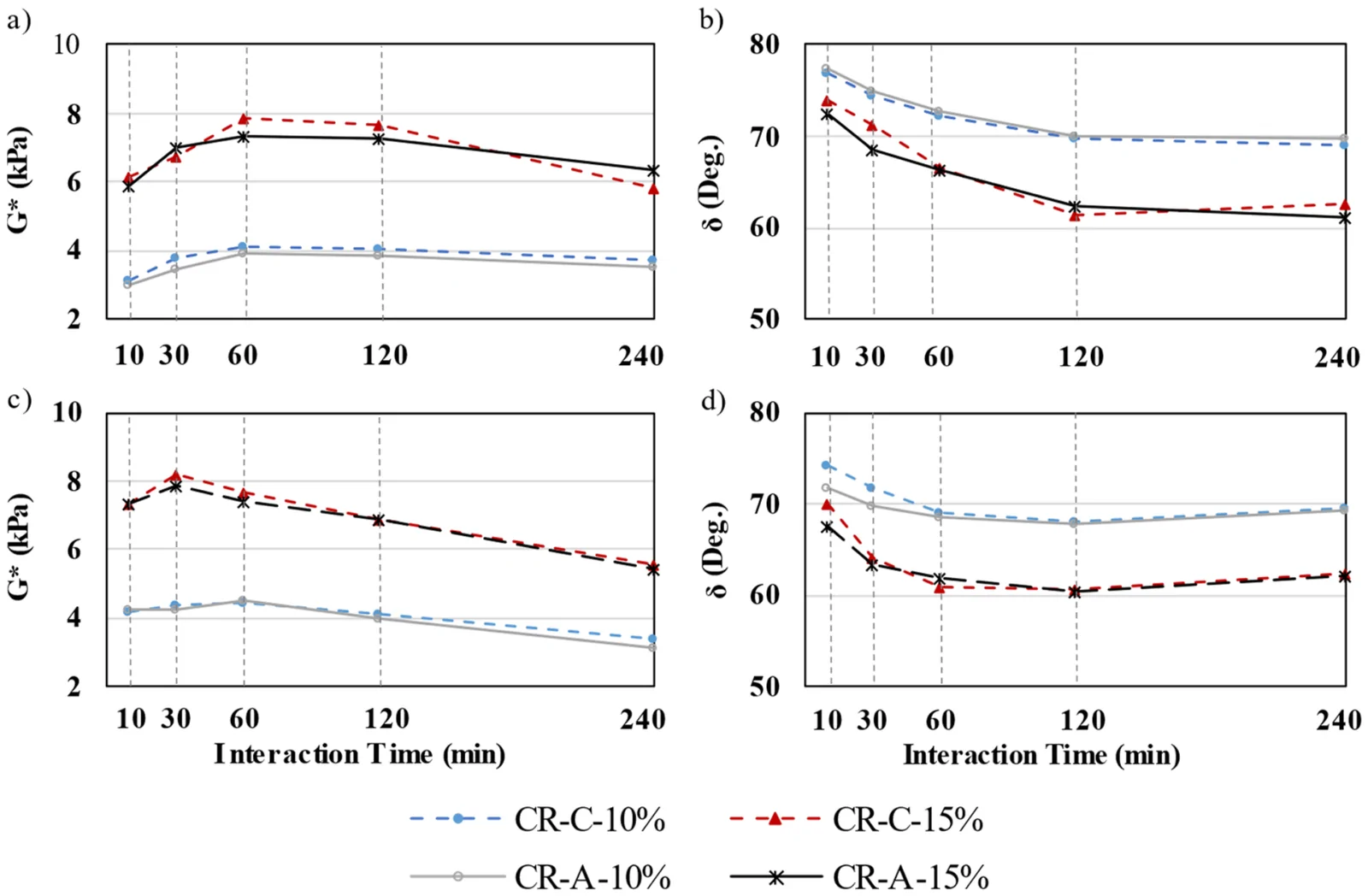
Evolution of Rheological Properties with Interaction Time for Investigated CRM Binders: (**a**) G* for 170 °C-Based CRM, (**b**) δ for 170 °C-Based CRM, (**c**) G* for 200 °C-Based CRM, (**d**) δ for 200 °C-Based CRM.

Figure 6 shows that rheological evolution depended strongly on CR content and interaction temperature. For the 10% CRM binders, G* increased to a maximum at approximately 60 min and then declined toward 240 min. Although the 200 °C binders developed higher early-stage stiffness, their subsequent reduction was greater, resulting in lower G* than the corresponding 170 °C binders after 240 min. For the 15% CRM binders, the maximum G* occurred at 30 min at 200 °C and at 60 min at 170 °C. Thus, the higher interaction temperature accelerated stiffness development but also produced a more pronounced decline during prolonged interaction.

Phase angle generally decreased with interaction time, indicating increased elastic response. Binders prepared at 200 °C exhibited lower δ during the early and intermediate stages, confirming faster rheological modification. However, for most 200 °C binders, δ reached a minimum near 120 min and increased slightly at 240 min, indicating a partial reversal toward more viscous behavior. In contrast, most 170 °C binders maintained decreasing or nearly stable δ values through 240 min. Overall, interaction at 200 °C accelerated rheological development but reduced long-term stability, whereas 170 °C produced slower and more sustained stiffness and elasticity.

Although CR content and interaction temperature were the dominant rheological factors, only minor differences were observed between cryogenic and ambient CRM binders. Both CR types exhibited similar G* and δ evolution trends, indicating that CR type had a secondary and condition-dependent effect. These slight differences may be associated with variations in particle morphology and surface characteristics, which can modestly influence CR dispersion and matrix interaction during the interaction process.

### 3.2. Effect of Interaction Time on BBS

**Figure 7:**
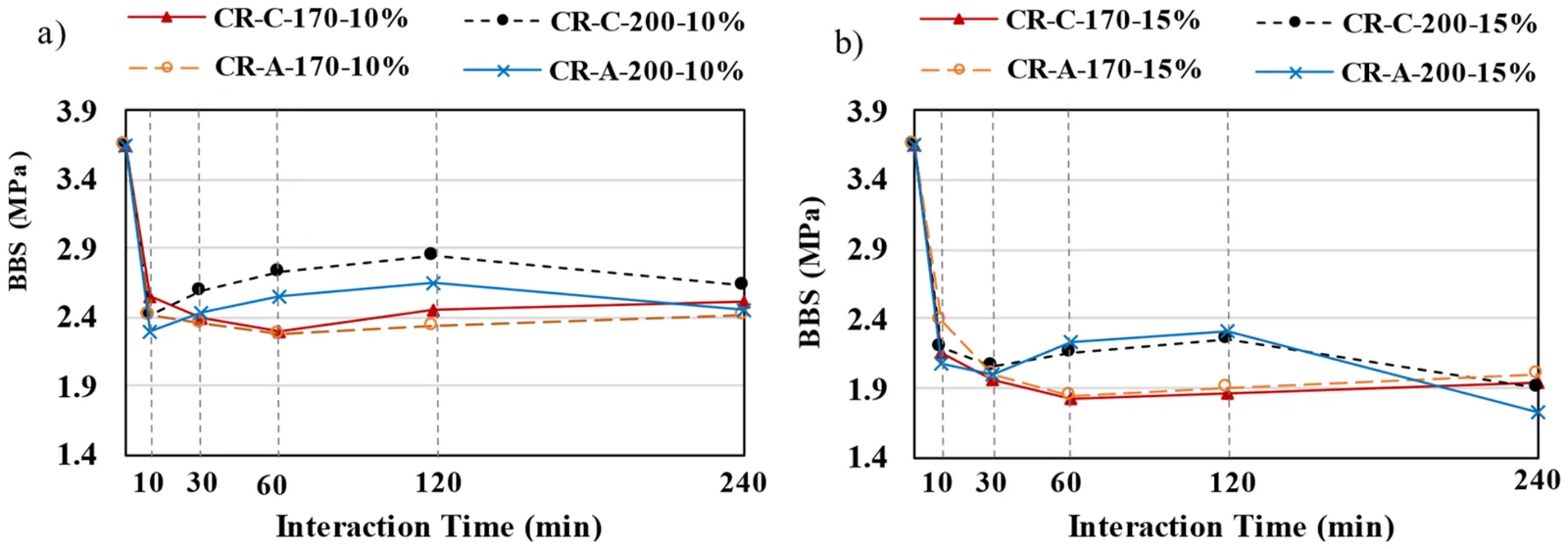
Effect of Interaction Time on Dry BBS of CRM Binders: (**a**) 10% CR, (**b**) 15% CR.

This section evaluates the bonding strength of the investigated CRM binders and its evolution throughout the CR–binder interaction process. The effects of CR type, CR content, interaction temperature, interaction time, and moisture conditioning on BBS are examined using the following results. Figure 7 presents the evolution of dry BBS during interaction at 170 and 200 °C. The unmodified base binder exhibited a dry BBS of 3.65 MPa. Following CR incorporation, an immediate reduction in BBS was observed after 10 min of interaction, with values ranging from 2.08 to 2.55 MPa depending on CR type, content, and interaction temperature. This initial reduction may be associated with incomplete CR dispersion and swelling during the early stage of interaction [46,47]. In addition, the presence of partially swollen rubber particles within the binder film may reduce the effective contact area between the asphalt binder and the substrate, thereby limiting bonding strength.

The evolution of BBS differed substantially between the two interaction temperatures, as shown in Figure 7. For the binders prepared at 170 °C, BBS generally decreased during the early stages of interaction and reached its minimum at approximately 60 min, followed by a gradual recovery toward 240 min. For example, the BBS of CR-C-170-10% decreased from 2.55 MPa at 10 min to 2.30 MPa at 60 min, before increasing to 2.45 and 2.51 MPa at 120 and 240 min, respectively. Similarly, CR-A-170-10% decreased from 2.42 MPa at 10 min to 2.27 MPa at 60 min and subsequently recovered to 2.41 MPa at 240 min. A comparable response was observed for the 15% binders. Between 120 and 240 min, all binders prepared at 170 °C exhibited a slight increase in BBS, indicating that interaction at the lower temperature resulted in a slower but more stable development of bonding strength during prolonged mixing.

In contrast, all binders prepared at 200 °C developed their maximum BBS at approximately 120 min, followed by a reduction at 240 min. At the 10% CR content, CR-C-200-10% increased from 2.42 MPa at 10 min to a maximum of 2.85 MPa at 120 min and then decreased to 2.64 MPa at 240 min, corresponding to a reduction of approximately 7.4%. Similarly, CR-A-200-10% reached 2.65 MPa at 120 min before decreasing by approximately 7.2% to 2.46 MPa. The decline was more pronounced at the 15% CR content. The BBS of CR-C-200-15% decreased from 2.25 MPa at 120 min to 1.90 MPa at 240 min, representing a reduction of approximately 15.6%, whereas CR-A-200-15% decreased from 2.30 to 1.72 MPa, corresponding to a reduction of approximately 25.2%. These results demonstrate that approximately 120 min represented the most favorable interaction period for the binders prepared at 200 °C, whereas extending the interaction to 240 min adversely affected bonding strength.

The observed BBS evolution may be explained by the competing mechanisms of CR swelling, dispersion, and degradation. During the initial interaction period, CR particles absorb the lighter fractions of the asphalt binder and begin to swell, while high-shear mixing promotes their dissolution throughout the binder. Improved dissolution and progressive swelling may contribute to the recovery and subsequent increase in BBS. At 200 °C, these mechanisms occur more rapidly, resulting in the development of maximum bonding strength at approximately 120 min. However, prolonged exposure to the higher interaction temperature may promote excessive degradation of the rubber-modified structure through depolymerization and devulcanization, which may contribute to the reduction in BBS between 120 and 240 min. This interpretation is supported by the phase angle results, for which δ increased consistently between 120 and 240 min. The increase in δ indicates a shift toward a more viscous and less elastic response, consistent with the partial deterioration of the CR-modified binder structure during prolonged high-temperature interaction. In comparison, interaction at 170 °C likely slowed both swelling and degradation, resulting in a more gradual and stable development of bonding strength.

CR content exerted a pronounced influence on BBS. The binders containing 10% CR consistently exhibited higher bonding strength than the corresponding 15% binders. Across the investigated conditions, the average dry BBS values were approximately 2.48 and 2.04 MPa for the 10% and 15% CR contents, respectively. The lower BBS at 15% CR may be attributed to the greater concentration of rubber particles, which increases binder viscosity and may reduce binder wetting and the effective contact area with the substrate. A Type III factorial ANOVA was used because the experimental program included multiple categorical factors (CR type, CR content, interaction temperature, and interaction time), as well as potential interactions among these factors. This approach enabled the effect of each factor to be evaluated after accounting for all other main and interaction effects included in the model, which was particularly important because the influence of one parameter could depend on the level of another. The ANOVA results presented in Table 3 confirmed that CR content was the dominant factor affecting dry BBS, with F = 213.37, *p* < 0.001, and a partial $\eta_{p}^{2}$ of 0.967. Interaction temperature and interaction time were also statistically significant, with *p* < 0.001 and *p* = 0.028, respectively, demonstrating that both processing conditions contributed significantly to the evolution of bonding strength.

The influence of CR type depended on CR content, as demonstrated by the significant CR type × CR content interaction (F = 5.68, *p* = 0.029). At the 10% CR content, the cryogenic binders consistently exhibited higher BBS than the corresponding ambient binders under the same temperature and interaction-time conditions. For example, at 120 min, CR-C-200-10% exhibited a BBS of 2.85 MPa compared with 2.65 MPa for CR-A-200-10%. At 240 min, the corresponding values were 2.64 and 2.46 MPa. However, the cryogenic advantage was not consistently maintained at the 15% CR content, particularly at 170 °C, where the ambient binders exhibited slightly higher values. Therefore, the effect of CR type cannot be interpreted independently of CR content. The significant temperature × time interaction (F = 11.75, *p* < 0.001) further confirms that the influence of interaction time strongly depended on the selected interaction temperature.

**Figure 8:**
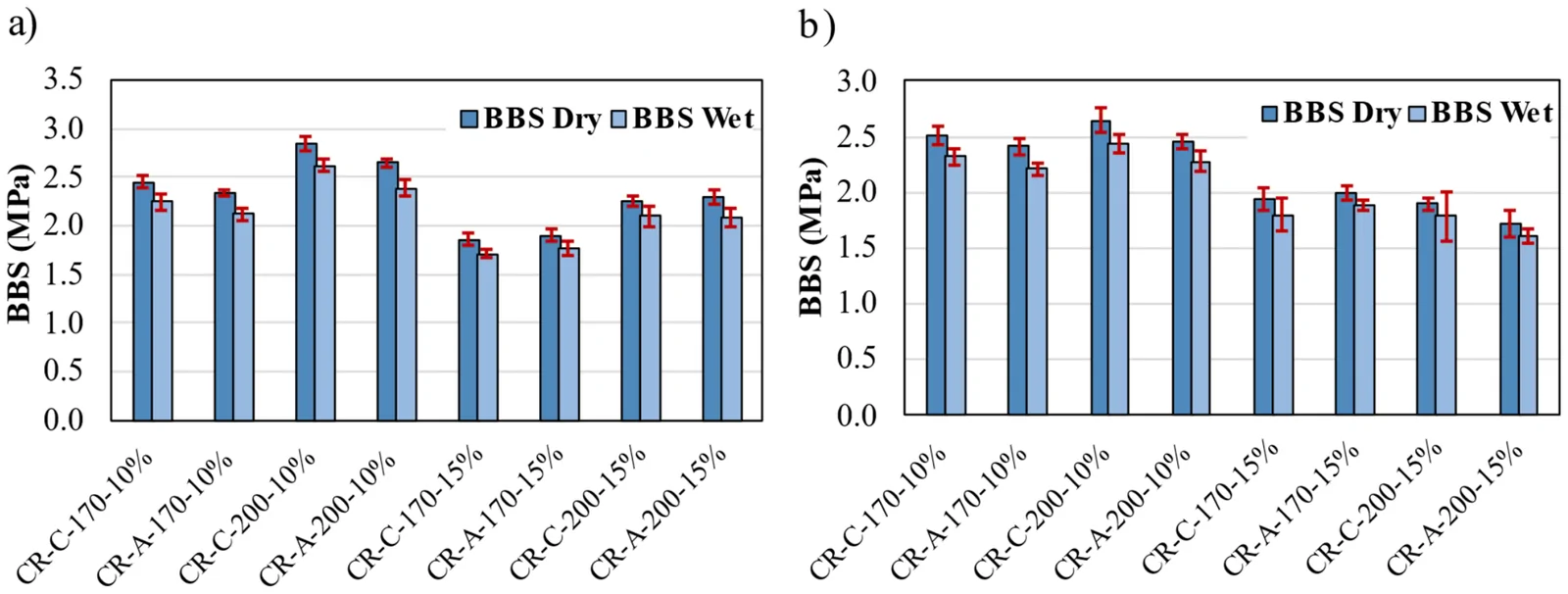
BBS Values at Dry and Wet Conditions: (**a**) 120 min Interaction Time, (**b**) 240 min Interaction Time.

Figure 8 compares binders conditioned for 120 and 240 min at dry and wet-conditioned states. Wet conditioning reduced the BBS of all binders; however, the CRM binders exhibited substantially lower moisture susceptibility than the base binder. The base binder decreased from 3.65 MPa under dry conditions to 2.78 MPa after 72 h of moisture conditioning, corresponding to a reduction of approximately 23.8%. In comparison, the moisture-induced BBS reductions of the CRM binders ranged from approximately 6.7% to 9.8% at 120 min and from 5.8% to 8.3% at 240 min. The average reductions were approximately 8.3% and 7.0% for the 120- and 240-min CRM binders, respectively. Thus, although CR incorporation reduced the absolute BBS relative to the base binder, it substantially improved the retention of bonding strength after moisture conditioning. This improved moisture resistance may be attributed to rubber-particle swelling, which increases binder viscosity and forms a robust physical barrier limiting water penetration, combined with the enhanced elastic recovery and cohesion of the cross-linked rubber network, which resists water-induced stripping and bond release.

At 120 min, CR-C-200-10% exhibited the highest dry and wet BBS values of 2.85 and 2.62 MPa, respectively. The same binder also maintained the highest values at 240 min, with dry and wet BBS values of 2.64 and 2.44 MPa, respectively. Nevertheless, the comparison between 120 and 240 min confirms that prolonged interaction was not beneficial for the binders prepared at 200 °C. Conversely, the binders prepared at 170 °C exhibited slight improvements in both dry and wet BBS between 120 and 240 min. Overall, the results demonstrate that bonding performance was governed by the combined effects of CR content, CR type, interaction temperature, and interaction time. An interaction period of approximately 120 min was most favorable at 200 °C, whereas the binders prepared at 170 °C exhibited a slower but more stable bonding-strength evolution during prolonged interaction.

### 3.3. Friction and Surface Texture Performance of CRM-Based HFST Systems

Before evaluating the polishing performance of the HFST systems, it should be noted that a binder application rate of 0.38 gal/yd^2^ was previously used in the study conducted by Aboelela et al. (2025) [11]. Although satisfactory performance was achieved for specimens incorporating calcined bauxite aggregate, the rhyolite-based specimens experienced severe aggregate loss during polishing despite using the same aggregate gradation and binder application rate [11]. Based on these observations, the binder application rate for the rhyolite specimens in the present study was increased to 0.44 gal/y^2^ to improve aggregate embedment and retention. The following discussion focuses on the HFST systems produced using the CRM binders prepared for 240 min of interaction.

**Figure 9:**
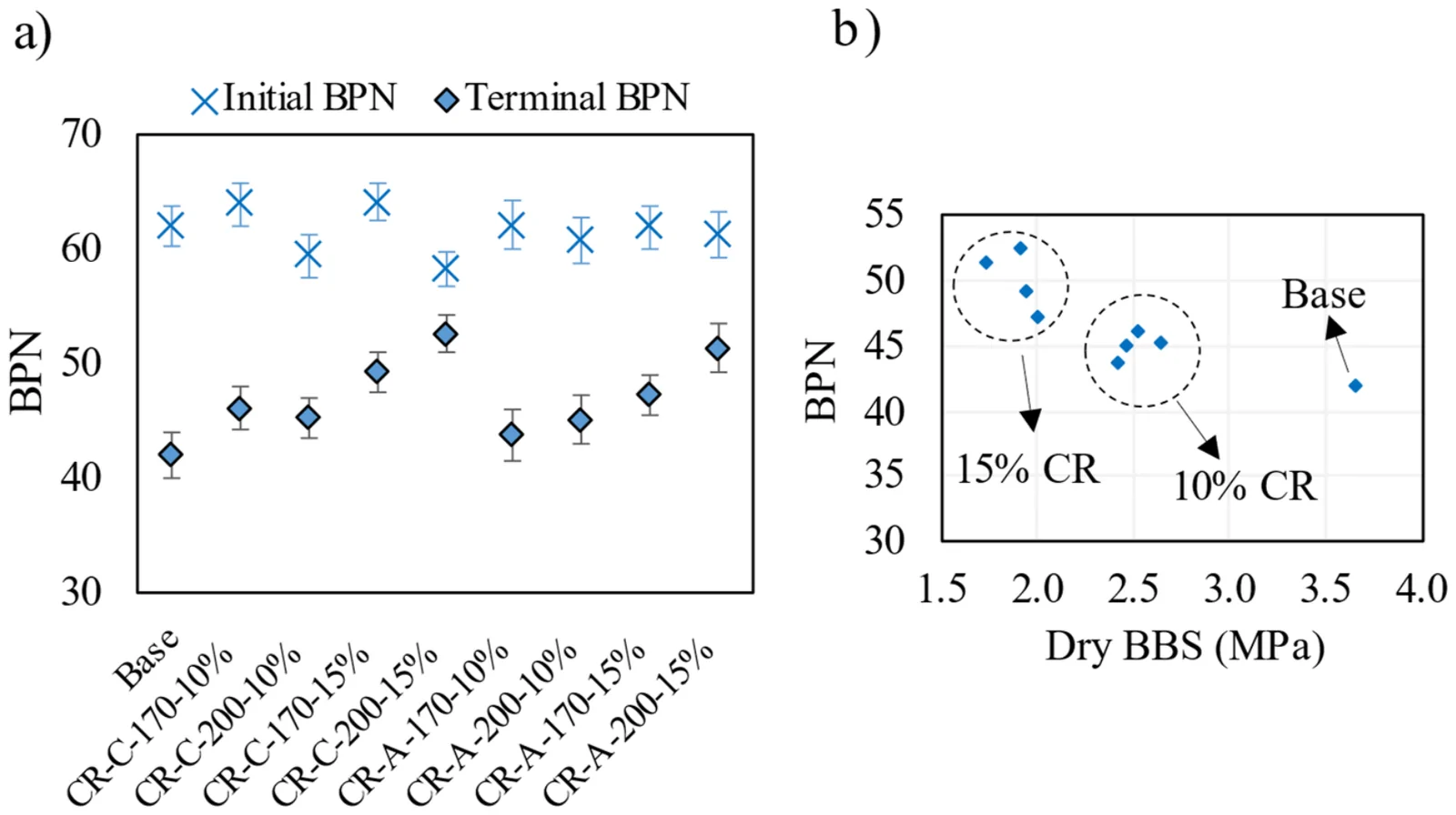
Friction Performance and Bonding Behavior of CRM-Based HFST Systems: (**a**) Initial and Terminal BPN after PSV Polishing and (**b**) Relationship between Terminal BPN and Dry BBS.

Figure 9a shows that the initial BPN values of the CRM systems ranged from 58.2 to 64.0 and were generally comparable to the base-binder value of 61.9. Thus, the initial surface friction was relatively similar across the investigated systems, whereas clear differences developed during polishing. The base-binder system exhibited the lowest terminal BPN of 42.0 and the greatest loss of 32.1%, while the CRM systems retained terminal values between 43.7 and 52.5. The 15% CRM systems consistently outperformed the corresponding 10% systems, suggesting greater resistance to polishing-induced friction deterioration and aggregate movement. CR-C-200-15% provided the best performance, retaining a terminal BPN of 52.5 with only 9.8% loss, followed by CR-A-200-15% with a terminal BPN of 51.3 and a 16.0% loss. The improved performance of the 15% systems indicates that the increased stiffness and elasticity associated with higher CR content likely enhanced aggregate stability and friction retention under repeated polishing.

Figure 9b demonstrates that terminal BPN did not increase with dry BBS. The base binder exhibited the highest dry BBS but the lowest terminal BPN, while the 15% CRM binders exhibited the lowest BBS range yet retained the highest BPN values. Thus, increasing CR content reduced the measured tensile bonding strength but improved resistance to aggregate loss and friction deterioration during polishing. This apparent difference reflects the distinct loading mechanisms represented by the two evaluations. BBS measures tensile resistance at the aggregate–binder interface, whereas polishing subjects embedded aggregate particles to repeated shear, rotation, and displacement. Therefore, a higher BBS does not necessarily provide greater resistance to polishing-induced aggregate movement. These results indicate that sufficient bonding is required to maintain aggregate attachment, but BBS alone does not govern HFST durability. Once an adequate bonding level and embedment are achieved, the increased stiffness and elasticity produced by the higher CR content can improve stress distribution, restrict aggregate rotation, and reduce progressive dislodgement. Accordingly, the improved BPN retention of the 15% CRM systems suggests that optimization should balance bonding strength with the rheological properties needed to resist shear-induced aggregate loss rather than focus solely on maximizing dry BBS.

**Figure 10:**
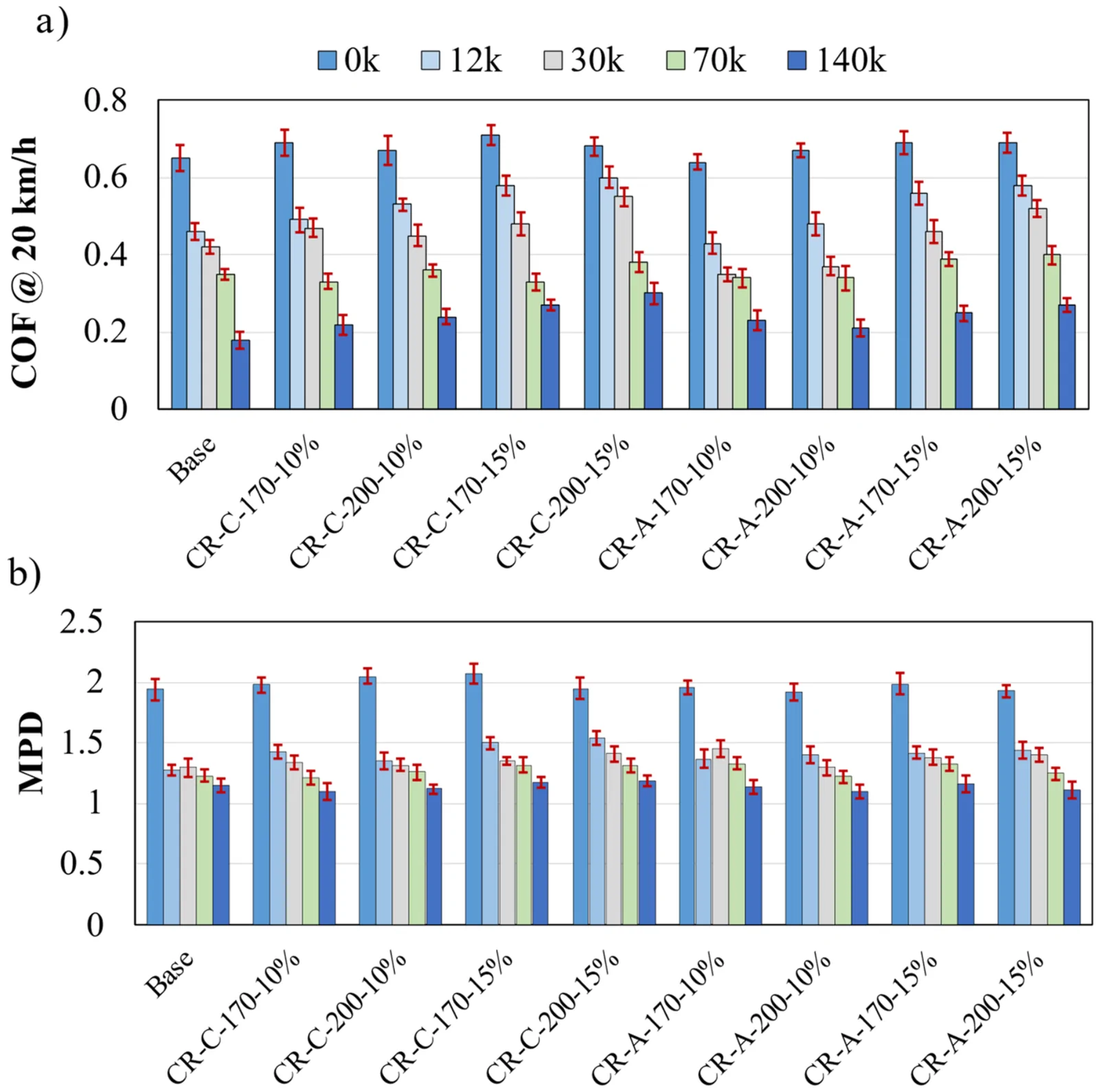
Effect of Polishing Cycles on (**a**) COF20 and (**b**) MPD.

Figure 10 presents the evolution of COF20 and MPD during accelerated polishing. All specimens exhibited a progressive reduction in both friction and macrotexture with increasing polishing cycles, with the greatest decline generally occurring during the early stage (0–12k cycles) as loosely retained particles were removed and aggregate asperities were exposed. After 140k cycles, the base binder exhibited the greatest overall deterioration, with a COF loss of 72.3%, compared with losses ranging from 55.9% to 68.7% among the CRM systems. The CRM systems containing 15% CR generally retained higher terminal COF and MPD values than the corresponding 10% systems, with CR-C-200-15% providing the best overall retention (COF loss of 55.9%).

**Figure 11:**
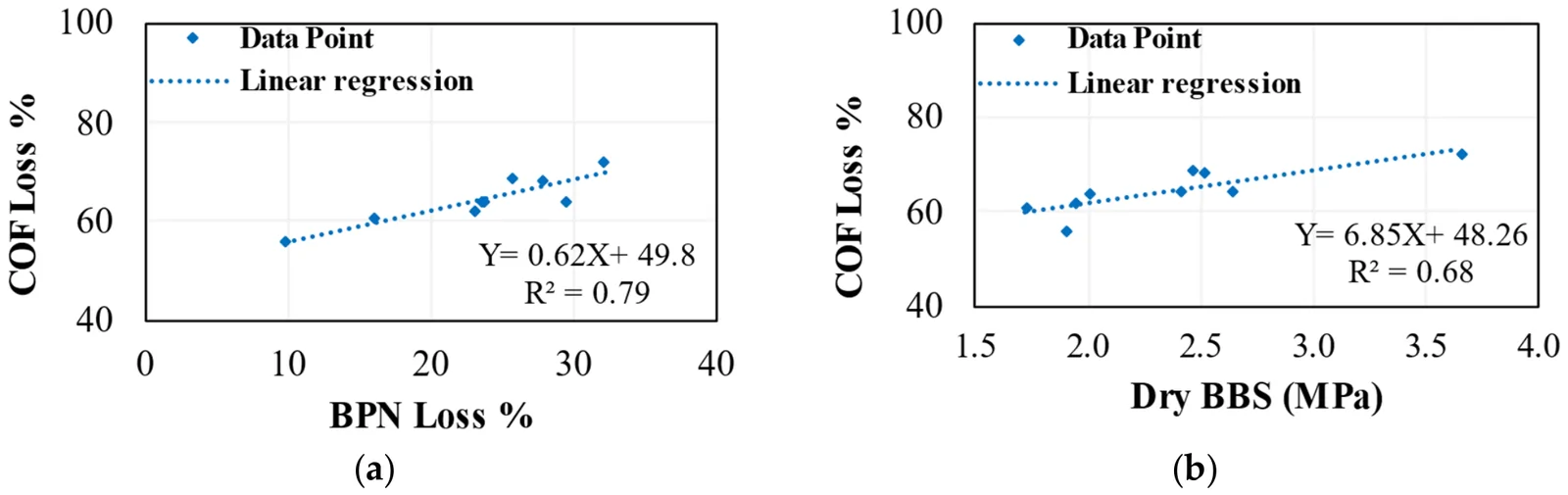
Relationship Between COF Loss and (**a**) BPN Loss and (**b**) BBS in Dry Condition.

Figure 11a shows a strong positive relationship between BPN loss and COF loss (R^2^ = 0.79), indicating that the BPT and DFT produced generally consistent rankings of polishing resistance. Systems with lower BPN loss also tended to retain higher COF, particularly those containing 15% CR. Additionally, Figure 11b shows a moderate positive relationship between BBS and COF loss (R^2^ = 0.68). This trend does not indicate that greater bonding strength caused greater friction loss; rather, it confirms that BBS alone was insufficient to predict HFST performance. The base binder exhibited the highest dry BBS but the greatest COF loss, whereas the 15% CRM binders developed lower BBS yet generally retained better friction. BBS evaluates tensile resistance at the aggregate–binder interface, while accelerated polishing subjects embedded particles to repeated shear, rotation, and displacement. Therefore, adequate bonding is necessary to prevent premature aggregate dislodgement, but long-term friction retention also depends on binder stiffness, elasticity, aggregate embedment, binder-film thickness, and resistance to shear deformation. These results indicate that CRM-based HFST optimization should balance bonding strength with rheological properties rather than rely solely on maximizing dry BBS.

#### 3.3.1. Effect of Interaction Time on Friction Performance Through BBS

**Figure 12:**
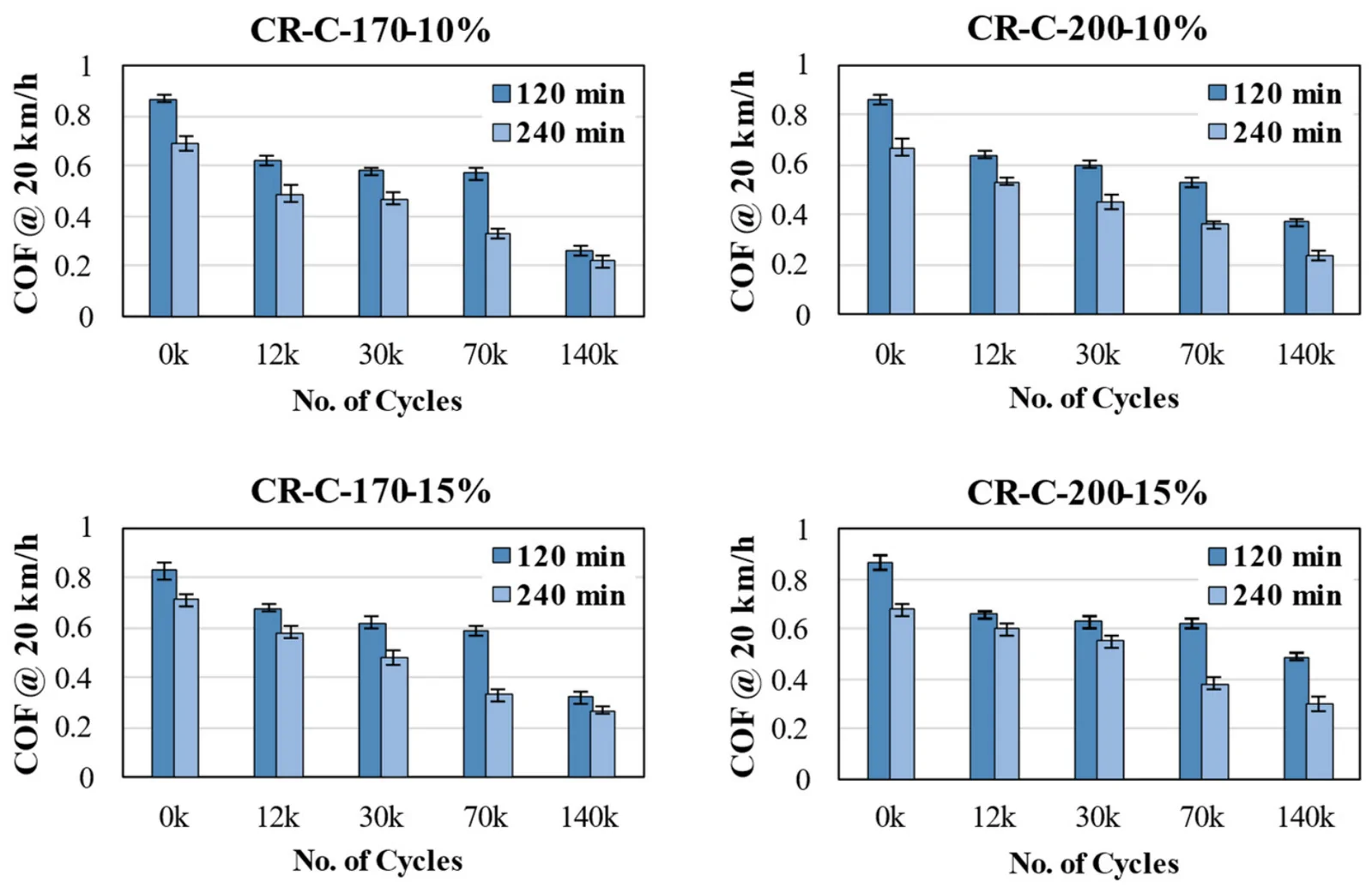
Comparison of COF at 20 km/h for cryogenic CRM binders prepared at interaction times of 120 and 240 min under accelerated polishing.

**Figure 13:**
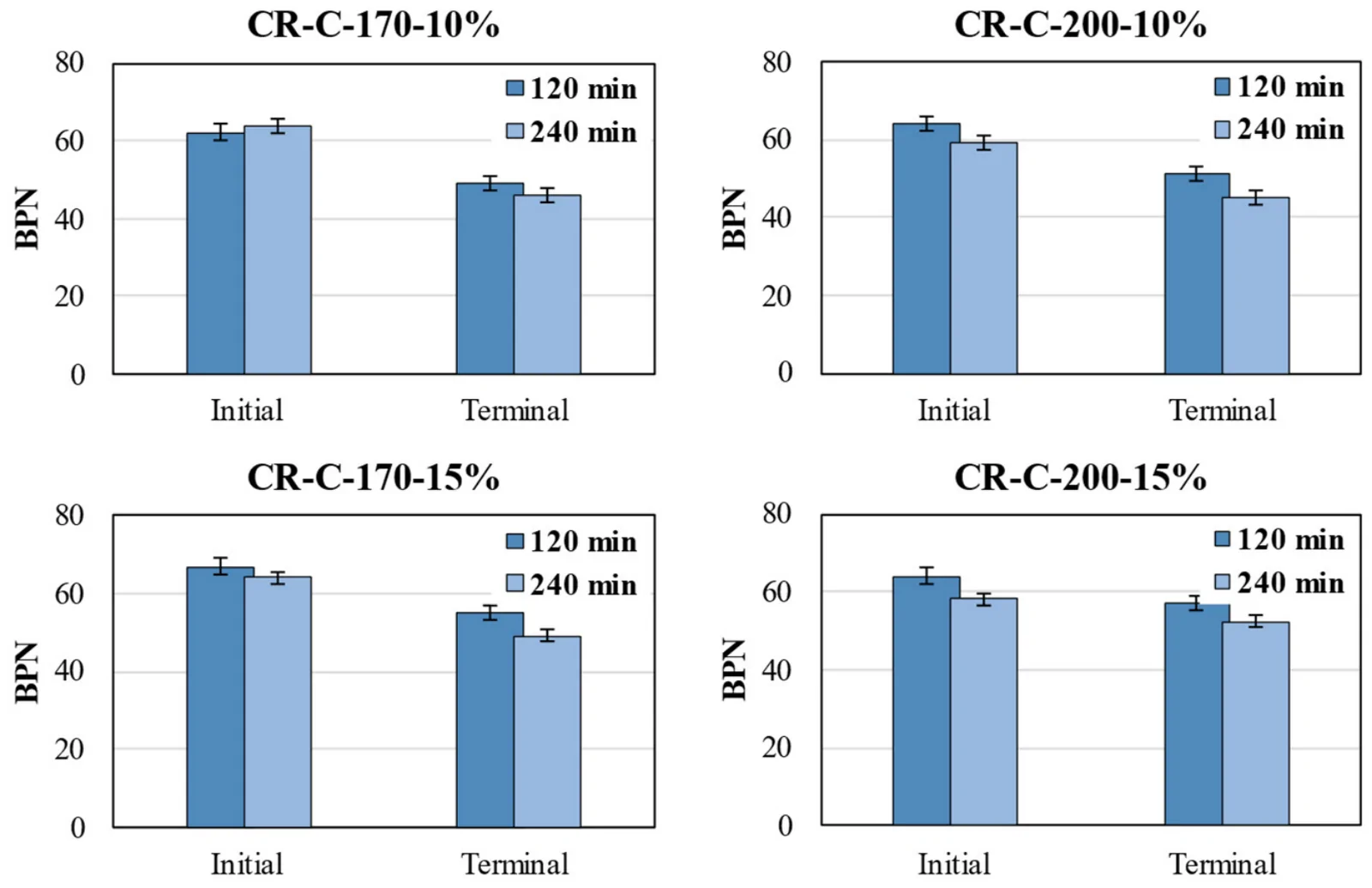
Comparison of BPN values for cryogenic CRM binders prepared at interaction times of 120 and 240 min under PSV polishing.

This section evaluates how interaction-time-dependent changes in BBS influence HFST friction performance by comparing identical cryogenic CRM formulations prepared for 120 and 240 min. The cryogenic CRM systems were selected for this comparison because they generally provided better friction retention than the corresponding ambient systems after 240 min of interaction. Figure 12 and Figure 13 compare the COF and BPN performance of cryogenic binders prepared for 120 and 240 min to clarify how interaction-time-dependent changes in BBS influenced HFST durability.

The effect of bonding was most evident for binders prepared at 200 °C. Increasing the interaction time from 120 to 240 min reduced dry BBS from 2.85 to 2.64 MPa for CR-C-200-10% and from 2.25 to 1.90 MPa for CR-C-200-15%. These reductions were accompanied by lower terminal COF values, decreasing from 0.37 to 0.24 and from 0.49 to 0.30, respectively. Terminal BPN also decreased from 51.0 to 45.2 for the 10% system and from 57.0 to 52.5 for the 15% system. Thus, within the same formulation at 200 °C, stronger bonding at 120 min corresponded to improved friction retention, indicating that the BBS developed during interaction contributed to limiting aggregate movement and polishing-induced deterioration.

In contrast, the 170 °C binders exhibited only slight increases in BBS between 120 and 240 min. BBS at dry condition increased from 2.45 to 2.51 MPa for CR-C-170-10% and from 1.86 to 1.94 MPa for CR-C-170-15%, while terminal COF and BPN decreased. Therefore, the relationship between BBS and HFST performance was not consistent at 170 °C. This response confirms that small changes in bonding strength alone cannot explain friction durability when other binder characteristics, including stiffness, elasticity, film condition, and resistance to shear deformation, also change with interaction time.

The 120-min systems maintained relatively similar COF values through approximately 70k polishing cycles, after which greater differentiation developed. The pronounced friction loss between 70k and 140k cycles indicates that prolonged polishing provided a clearer assessment of the ability of each binder to restrict aggregate rotation and displacement. CR-C-200-15% exhibited the greatest late-stage friction retention, demonstrating that effective HFST optimization requires sufficient bonding combined with the rheological properties provided by higher CR content.

These findings clarify the apparent positive relationship between dry BBS and COF loss obtained when all 240-min binders were evaluated together. That overall relationship was influenced by differences in CR content and rheology and did not represent a direct causal effect of BBS. When the same 200 °C formulation was compared at different interaction times, higher BBS was associated with better HFST performance. However, the 170 °C results confirm that maximizing BBS alone is insufficient. Accordingly, bonding optimization should target the interaction condition that develops adequate and moisture-resistant adhesion while preserving the stiffness and elasticity required for aggregate retention. For the investigated cryogenic binders, an interaction time of approximately 120 min at 200 °C provided the most favorable balance between BBS development and friction durability.

#### 3.3.2. Comparison Between Rhyolite-Based and Calcined Bauxite-Based HFSTs

This section presents a comprehensive comparison between the performance of rhyolite-based HFST systems investigated in the current study and the calcined bauxite and rhyolite-based HFST systems reported by Roshan and Abdelrahman (2025) [48] and Aboelela et al. (2025) [11]. The comparison considers the combined effects of aggregate type, binder type, and binder application rate on aggregate retention and long-term friction performance, as evaluated using terminal COF20 and BPN values after accelerated polishing. As shown in Table 4, the calcined bauxite systems exhibited satisfactory polishing resistance, with terminal COF20 values of 0.53–0.66 and BPN values of 56–74. In contrast, the previous rhyolite systems prepared at 0.38 gal/yd^2^ experienced premature aggregate loss. Failure occurred at approximately 15k cycles for CR-C-10%, 28k cycles for the base binder, and 60–61k cycles for CR-C-15% [11]. These results indicate that the application rate used successfully with calcined bauxite did not provide sufficient embedment and retention for the rhyolite aggregate. In the present study, the rhyolite binder application rate was increased to 0.44 gal/y^2^. This modification eliminated premature failure and enabled all systems to complete the 140k-cycle polishing program. Among the CRM systems, CR-C-200-15% provided the best terminal performance, with a COF20 of 0.30 and a BPN of 52.5, followed by CR-C-170-15%. The 15% CRM systems generally outperformed the corresponding 10% systems, indicating that aggregate retention depended not only on absolute BBS but also on binder stiffness, elasticity, and resistance to aggregate movement. Although the increased binder rate substantially improved the durability of the rhyolite systems, their terminal COF20 values remained lower than those of the calcined bauxite systems. Therefore, increasing the application rate can improve aggregate embedment and prevent premature loss, but it cannot fully compensate for the lower polishing resistance of rhyolite. Overall, the results demonstrate that the binder rate should be selected according to both aggregate characteristics and binder rheology rather than applied uniformly to all HFST systems.

## 4. Conclusions and Recommendations

This study investigated the optimization of the bonding behavior of crumb rubber-modified (CRM) binders by evaluating the evolution of binder bonding strength (BBS) under different interaction conditions and assessing the influence of the optimized CRM binders on the accelerated-polishing performance of rhyolite-based high-friction surface treatment (HFST) systems. The main findings are:

BBS evolution was strongly dependent on interaction temperature. At 170 °C, BBS generally recovered toward 240 min, whereas at 200 °C, BBS generally reached its highest level near 120 min and decreased thereafter.

The interaction parameters significantly influenced BBS evolution. ANOVA identified CR content as the dominant factor affecting BBS (F = 213.37, *p* < 0.001, $\eta_{p}^{2}$ = 0.967), while interaction temperature, interaction time, CR type × CR content, and temperature × time were also significant.

Although CRM binders exhibited lower dry BBS than the base binder, they showed substantially greater moisture resistance, with BBS reductions of only 5.8–9.8% compared with 23.8% for the base binder.

HFST durability was not governed by maximum BBS alone. The 15% CRM binders generally exhibited lower BBS but retained higher terminal BPN, COF, and MPD than the corresponding 10% binders, demonstrating the importance of balancing bonding strength with binder stiffness and elasticity.

Cryogenic CRM binders generally exhibited better friction retention than the corresponding ambient CRM binders, particularly at the higher CR content. For the cryogenic formulations specifically evaluated at both 120 and 240 min, the 120-min condition at 200 °C produced higher BBS and higher terminal COF and BPN than the corresponding 240-min condition. At 170 °C, changes in BBS between 120 and 240 min were not consistently reflected in friction performance.

Finally, the results demonstrate that bonding strength alone does not govern HFST durability. However, for the investigated CRM binders, interaction-time-dependent changes in BBS affected friction retention, emphasizing the importance of optimizing interaction conditions while maintaining sufficient bonding, stiffness, and elasticity for CRM-based HFST applications. Future research should investigate the chemical and morphological evolution of CRM binders during the interaction process and quantify its relationship with bonding-strength development.

## Figures and Tables

**Table 1:** Characterizations of Base Asphalt.

Property	Value	Specifications
Viscosity @ 170 °C (Pa·s.)	0.45	ASTM D4402 [30]
Softening Point (°C)	72	ASTM D36 [31]
Penetration at 25 °C (0.1 mm)	19.4	ASTM D5 [32]
Performance Grade	PG 88-16	ASTM D6373 [33]

**Table 2:** Physical Characterizations of Investigated Aggregates.

Property		Calcined Bauxite	Rhyolite	Specifications
Bulk Specific Gravity (Gsb)		3.25	2.56	ASTM C128-22 [34]
Water Absorption (%)		2.5	0.9	AASHTO T85-22 [35]
LAA (%)		16	17	AASHTO T96 [36]
Gradation Passing (%)	#4	100 (Min 100)		AASHTO T27 [37]
	#6	95 (Min 90)		
	#16	2.5 (Max 5)		

**Table 3:** ANOVA Results for the Effects of CR Characteristics and Interaction Conditions on BBS.

Factor	F-Value	*p*-Value	Partial $\eta_{p}^{2}$	Significant
CR content	213.37	<0.001	0.967	Yes
Interaction temperature	18.96	<0.001	0.724	Yes
Interaction time	3.54	0.028	0.605	Yes
CR type × CR %	5.68	0.029	0.440	Yes
Temperature × time	11.75	<0.001	0.859	Yes

**Table 4:** Comparison of COF20 and BPN Values for Rhyolite- and Calcined Bauxite-Based HFSTs.

Binder Type	Aggregate Type	Binder Rate (Gallon/yd^2^)	COF20 (140k)	Terminal BPN	Source
Epoxy Resin	Calcined Bauxite	0.3 to 0.38	0.62	74	[48]
	Rhyolite		0.38	49	
Base Binder	Calcined Bauxite	0.38	0.60	65	[11]
	Rhyolite	0.38	(Failure at 28k)	N/A	
	Rhyolite	0.44	0.18	42	Current study
CR-C-170-10% (240 min)	Calcined Bauxite	0.38	0.51	56	[11]
	Rhyolite	0.38	(Failure at 15k)	N/A	
	Rhyolite	0.44	0.22	46.0	Current study
CR-C-200-10% (240 min)	Calcined Bauxite	0.38	0.53	65.7	[11]
	Rhyolite	0.38	(Failure at 15k)	N/A	
	Rhyolite	0.44	0.24	45.2	Current study
CR-C-170-15% (240 min)	Calcined Bauxite	0.38	0.62	70.6	[11]
	Rhyolite	0.38	(Failure at 61k)	N/A	
	Rhyolite	0.44	0.27	49.2	Current study
CR-C-200-15% (240 min)	Calcined Bauxite	0.38	0.66	71.6	[11]
	Rhyolite	0.38	(Failure at 60k)	N/A	
	Rhyolite	0.44	0.30	52.5	Current study

## Data Availability

The original contributions presented in the study are included in the article, further inquiries can be directed to the corresponding author.
